# Localized Bioimpedance Measurements with the MAX3000x Integrated Circuit: Characterization and Demonstration

**DOI:** 10.3390/s21093013

**Published:** 2021-04-25

**Authors:** Shelby Critcher, Todd J. Freeborn

**Affiliations:** Department of Electrical and Computer Engineering, The University of Alabama, Tuscaloosa, AL 35487, USA; tjfreeborn1@eng.ua.edu

**Keywords:** bioimpedance, localized tissues, Maxim MAX3000x, multi-frequency, resistance/reactance

## Abstract

The commercial availability of integrated circuits with bioimpedance sensing functionality is advancing the opportunity for practical wearable systems that monitor the electrical impedance properties of tissues to identify physiological features in support of health-focused applications. This technical note characterizes the performance of the MAX3000x (resistance/reactance accuracy, power modes, filtering, gains) and is available for on-board processing (electrode detection) for localized bioimpedance measurements. Measurements of discrete impedances that are representative of localized tissue bioimpedance support that this IC has a relative error of <10% for the resistance component of complex impedance measurements, but can also measure relative alterations in the 250 mΩ range. The application of the MAX3000x for monitoring localized bicep tissues during activity is presented to highlight its functionality, as well as its limitations, for multi-frequency measurements. This device is a very-small-form-factor single-chip solution for measuring multi-frequency bioimpedance with significant on-board processing with potential for wearable applications.

## 1. Introduction

The passive electrical impedance of biological tissues, often referred to as tissue bioimpedance, is being widely investigated as a technique for identifying physiological features in support of health-focused applications [1]. A non-exhaustive sample of applications under active investigation includes identifying acute tissue injury [2,3] and skeletal muscle fatigue [4,5], assessing neuromuscular disorders [6], tracking fluid shifts during dialysis [7], assessing joint health [8,9], blood pressure monitoring [10], and respiratory monitoring [11]. In each of these applications, tissue bioimpedance is related to the tissue fluid, type, structure, and geometry. Changes in each feature are expected to alter the tissue impedance and potentially serve as an indicator of the underlying mechanism of change (e.g., fluid shift, tissue damage, tissue swelling) motivating their study. Additionally, bioimpedance measurements are non-invasive (when collected using surface electrodes interfaced with the skin site over the target tissue), use low energy, and require simpler circuitry (and potentially lower costs) than other tissue characterization techniques (e.g., ultrasound, magnetic resonance imaging). The potential for physiological monitoring using low-cost instrumentation provides a strong motivation for the continued research of health-focused bioimpedance monitoring and applications.

Regardless of the application, measurement of tissue bioimpedance requires an interface with the targeted tissue, circuitry to excite the tissue and measure the response, and processing of measurements to identify relevant features for the specific application. Each of these high-level aspects of bioimpedance measurements is an area of active research and development. At the interface between circuitry and tissue, groups are exploring dry electrodes [12,13] and their impact on measurement quality. Beyond materials, groups are developing configurations and recommendations for electrode placement to support improvement of the measurement of muscle [14] and radial arteries [15]. There is also active research into different measurement schemes for bioimpedance instrumentation (e.g., modulus, phase, impedance-bridge, oscillator, quadrature, etc.), with Naranjo-Hernández et al. providing a comprehensive review for interested readers [1]. Next, advances in measurement schemes and required circuitry are being pursued on multiple fronts, including correction techniques [16] and fabrication of integrated circuits (ICs) to implement these measurements [17]. Beyond data collection, there are efforts to explore how to process and identify features to support health-focused applications: utilizing equivalent circuit representations and the associations of circuit parameters with tissue features [5], as well as multi-frequency time-series data [18].

Many research efforts are motivated by a drive to advance bioimpedance instrumentation from portable to wearable devices. This transition is expected to increase opportunities for continuous monitoring and health-focused applications. Commercially available integrated circuits (ICs) with dedicated bioimpedance functionality provide the opportunity for development of bioimpedance wearables without expertise in designing application-specific analog circuitry. This reduces the barrier for researchers and designers interested in implementing bioimpedance sensing for their studies or products. Available ICs with bioimpedance measurement functionality include the Analog Devices AD5933, Texas Instruments AFE4300, Analog Devices ADuCM350, and Maxim Integrated MAX30001/30002. Each of these ICs have different features and functionalities. Table 1 summarizes the important features (e.g., datasheet-specified frequency range, default supported electrode configurations, size, applications, required analog-front-end circuitry) and studies that have characterized these ICs. From the details in Table 1, all bioimpedance ICs have similar upper frequency measurement limits (64 to 131 kHz), but there is an order of magnitude of difference between their lower frequency measurement limits (80 Hz to 1 kHz). Table 1 also details the impedance range specified in the ICs’ datasheets (Imp. Range) where available, as well as the impedance range of bioimpedance measurements reported in the literature for these ICs (Lit. Imp. Range). Note that while the AD5933 datasheet [19] specifies a minimum measurable impedance of 1 kΩ, researchers have extended this range to <10 Ω [20]. To further highlight differences in these ICs, both the current excitation signal and measurement type are detailed in Table 1. Note that the MAX3000x is the only IC that implements a square-wave excitation signal to determine the measured impedance value. For applications focused on delivering the smallest devices possible, the package size and necessary additional circuitry are important design considerations. With those features in mind, the MAX3000x provides the smallest package size of the ICs given in Table 1. In addition, it does not require additional circuitry to expand the functionality to collect tetrapolar measurements or expand the impedance measurement range to include the range typical of localized tissues. While the AD5933 has been available since 2005 and has been the most investigated bioimpedance IC [18,20,21,22,23,24,25,26], it again requires an additional analog front end (AFE) for tetrapolar measurements [22,23] and to expand its measurement range [20].

As additional ICs with bioimpedance functionality become available (such as the MAX30001/30002 in 2019), researchers and designers benefit from the performance and operation characterization of these devices. These details support researchers’ evaluations of these devices and their appropriateness for their specific needs. While the MAX30001/30002 has been available since 2019 and has been utilized for preliminary studies on respiratory [27] and ECG monitoring [28,29], there are limited explorations of its measurement accuracy for values that are representative of localized tissues [30,31]. Additionally, the MAX30001/300002 supports wide-band multi-frequency measurements (125 Hz to 131 kHz) and tetrapolar measurements (without additional circuitry) in the smallest form factor (8 mm${}^{2}$ ball-grid array package) of the available bioimpedance ICs in Table 1. These features make this an attractive option for wearable systems, but require further investigation of performance. This provides the motivation for this technical note, which is to characterize the performance of the Maxim Integrated MAX30001/30002 for localized bioimpedance measurements.

In this technical note, the high-level operational theory of the MAX30001/30002 and its on-board processing will be detailed. Beyond the operational theory, the accuracy of the MAX30001’s impedance measurements was experimentally evaluated. Experimental results were collected from discrete circuits (with values representative of localized tissues) from a range of the MAX30001’s operating conditions (filtering, gains, excitation currents). These experimental measurements were compared with reference values collected using a Keysight E4990A precision impedance analyzer. This technical note expands on preliminary reports by Critcher and Freeborn on the resistance and reactance accuracy of this IC [30,31] in order to characterize the performance across the operational modes of this device. Finally, bioimpedance data collected during activity are presented to highlight the functionality, potential applications, and limitations of this device. The aim of this technical note is to support researchers interested in evaluating this sensing IC for localized bioimpedance measurements for potential adoption for their particular efforts.

## 2. MAX30001/30002 Bioimpedance Analog-Front-End Theory of Operation

**Figure 1:**
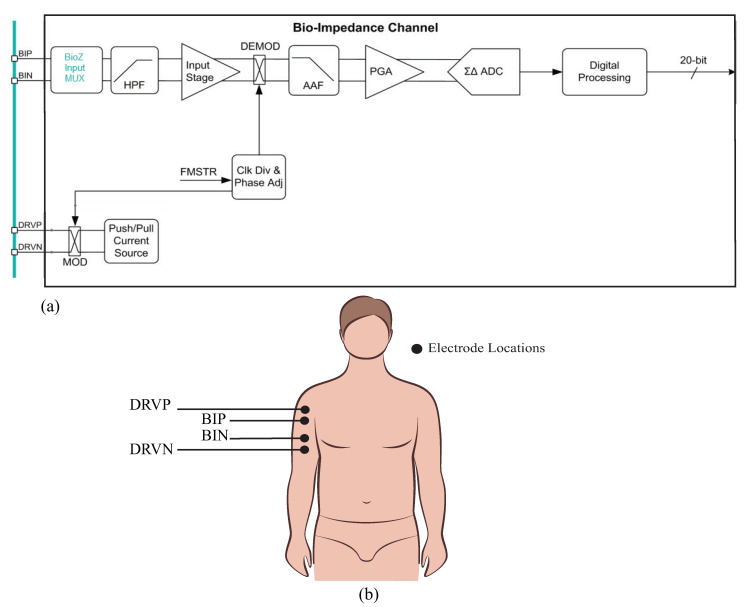
(**a**) MAX3000x bioimpedance channel outlining the high-level signal conditioning applied to the signal measured at the voltage sensing pins (BIP, BIN) and (**b**) sample tetrapolar interface of the MAX3000x bioimpedance pins (DRVP, DRVN) with a localized bicep tissue.

The MAX30001/30002 (collectively referred to as the MAX3000x for the remainder of this work) are single-chip integrated circuits with analog-front-end circuitry designed for bioimpedance measurements. The MAX30002 is exclusively a bioimpedance IC, while the MAX30001 also has analog-front-end circuitry for additional biopotential measurements. These ICs are capable of two-electrode (bipolar) and four-electrode (tetrapolar) measurements with a wide range of configurable features to customize the performance. The bioimpedance channel of this device is given in Figure 1. Operationally, these ICs generate a square-wave current excitation signal that is applied to the tissue/device under study. The positive (DRVP) and negative (DRVN) current source device pins, shown in Figure 1, apply this current to the tissue/device under study. The resulting voltage response to this current is monitored using the bioimpedance positive (BIP) and negative (BIN) device pins. While there are numerous approaches to determining the type of current excitation to apply (rectangular pulses, chirp waveforms, Gaussian waveforms, etc.), rectangular pulses are the simplest to generate; Min et al provided an overview of different excitation signals [39] for interested readers.

**Figure 2:**
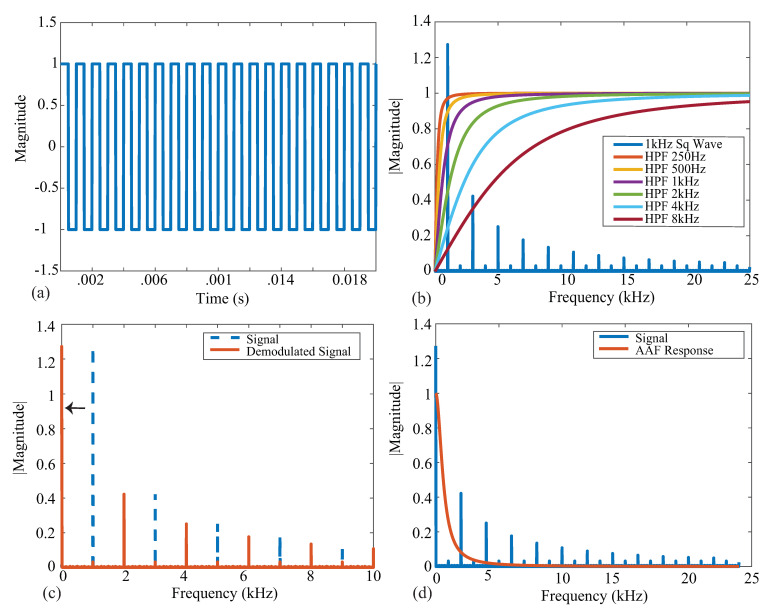
(**a**) Ideal square wave with an amplitude of 1 and a frequency of 1 kHz. (**b**) Single-sided frequency spectrum of a 1 kHz square wave (blue) plotted with ideal HPF magnitude responses with the corresponding cutoff frequencies. (**c**) Single-sided spectrum of a 1 kHz square wave (dashed blue) plotted with the demodulated (orange) signal. (**d**) Demodulated signal (blue) plotted with an AAF magnitude response (orange).

The voltage that is monitored by the MAX3000x (captured by the BIP and BIN inputs in Figure 1) is high-pass filtered (HPF), demodulated to DC, anti-alias filtered (AAF), amplified by a programmable gain amplifier (PGA), and converted into a digital representation using a ΣΔ analog-to-digital converter (ADC) for further digital processing (filter/decimation). After this conditioning and digital conversion, the sensed voltage is available for transfer to an external microcontroller (or equivalent device) through a serial peripheral interface (SPI). Based on the datasheet’s references to demodulation and configurable settings for the mixing frequency, it is hypothesized that this IC utilizes a quadrature demodulation [1,40,41] with a single multiplication. However, this hypothesis (and its implications for measuring resistance and reactance) will be explored in greater detail in the following sections. To visualize the excitation/measurement signal and the frequency spectrum throughout the signal conditioning stages of the bioimpedance channel, MATLAB simulations of ideal steps in this process are provided in Figure 2.

An ideal square wave with amplitude A=1, frequency f=1 kHz, and phase shift Θ=0 is given in Figure 2a, representing an ideal voltage response to a current excitation applied to a tissue/device. The single-sided frequency spectrum of this signal is given as the blue line in Figure 2b. The coefficient with the greatest magnitude is at a frequency of 1 kHz with odd order harmonics of decreasing amplitude, highlighting that the square wave causes a wide frequency band excitation. Calculating the impedance for only a single frequency requires the removal of the harmonic contributions, which is partially achieved with the analog high-pass filter at the input of the bioimpedance channel. The impacts of harmonics on bioimpedance measurements using square-wave current excitation have been explored by Min and Parve [40], with correction techniques proposed by Kubendran et al. [42] and Subhan and Ha [16].

The ideal frequency spectrum of the square-wave signal in comparison with the filter magnitude characteristics is given in Figure 2b. The square spectrum is shown in blue, and the high-pass filter magnitude responses with cutoff frequencies 250 Hz, 500 Hz, 1 kHz, 2 kHz, 4 kHz, and 8 kHz are shown. These filter responses were generated using a first-order high-pass transfer function given by:

(1) $$H\left(\omega \right)=\frac{s}{s+\omega_{c}}$$

where $\omega_{c}$ is the cutoff frequency in rad/s. These six frequencies were selected because they represent the available options of the MAX3000x. Notice that the higher the cutoff frequency of this filter, the greater the attenuation at the primary harmonic, which will impact the accuracy of the bioimpedance calculated from this attenuated voltage. Following the analog high-pass filter, an instrumentation amplifier (INA) with a programmable power mode amplifies the input voltage. This is shown in Figure 1 by the Input Stage block.

Next, the signal is demodulated to shift the signal of interest to DC (illustrated in Figure 2c). After demodulation, a second-order AAF with $f_{c}=600$ Hz is applied. To contrast the frequency spectrum of the demodulated signal with the magnitude characteristics of the AAF, both are shown in Figure 2d. The AAF will reduce the amplitude of the higher-order harmonics. Continuing through the signal conditioning blocks, a programmable gain amplifier (with user-selectable gains of 10 *v*/*v*, 20 *v*/*v*, 40 *v*/*v*, and 80 *v*/*v*) amplifies this signal. The specific gain configurations and their effects on localized bioimpedance measurements are explored in Section 3.5. After amplification, the signal is converted into its digital representation by a ΣΔ ADC, sampling at approximately 32 kHz. This digital value is decimated to yield measurements that can be collected at 32 or 64 samples per second (sps).

The digital representations outputted by the MAX3000x are converted into their impedance by:

(2) $$Z\left(\Omega \right)=\mathrm{ADC}\times \frac{V_{\mathrm{REF}}}{2^{19}\times \mathrm{CG}\_\mathrm{MAG}\times \mathrm{GAIN}}$$

where *Z* is the measured impedance, ADC is the internal analog-to-digital value of the sensed voltage, $V_{\mathrm{REF}}$ is the internal voltage reference, CG_MAG is the user-selected magnitude of the current excitation, and GAIN is the user-selected internal gain applied to the voltage measurement.

## 3. MAX3000x Performance Characterization

**Figure 3:**
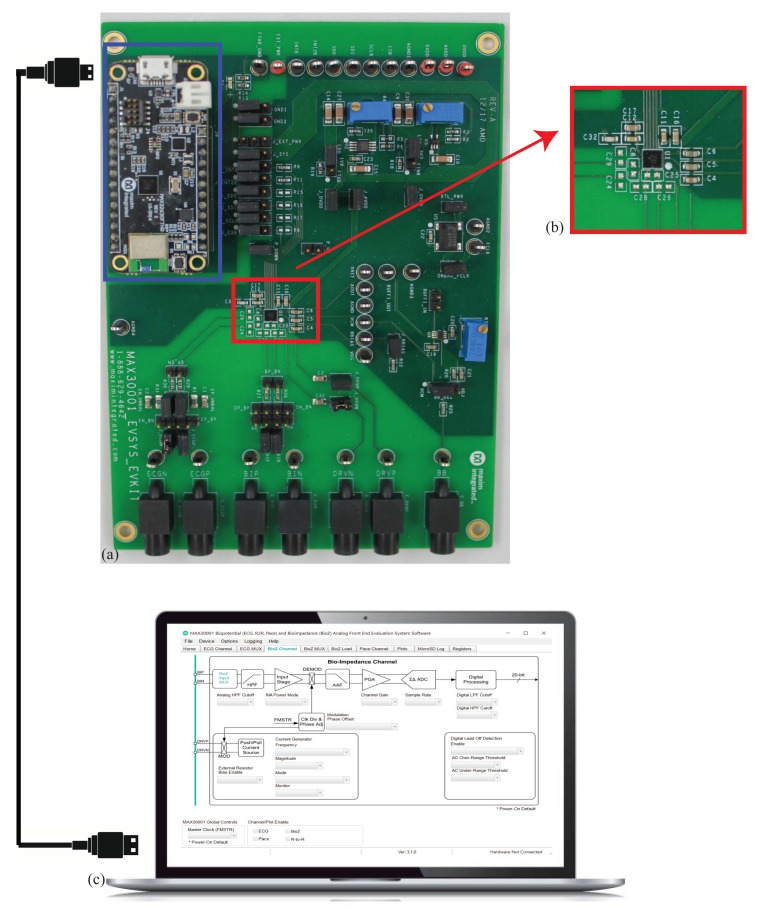
(**a**) MAX3000x development kit for control of (**b**) MAX30001 IC and peripheral circuitry (**c**) MAX3000x support software on a connected computer to control the bioimpedance channel interface.

To characterize the performance of the MAX3000x, an available development kit (MAX30001EVSYS) was utilized to collect measurements of discrete circuits and localized tissues. A sample development kit, shown in Figure 3, includes the MAX30001, power regulation circuitry, physical interfaces for connecting to an external device/tissue, and an MAX32630FTHR microcontroller. The on-board microcontroller provides communication/control over USB by a connected computer with necessary interfacing software provided by Maxim. This setup, which is detailed in Figure 3, provides rapid and convenient experimentation with the MAX30001 to configure/monitor register settings and collect measurements without hardware and firmware/software development.

**Figure 4:**
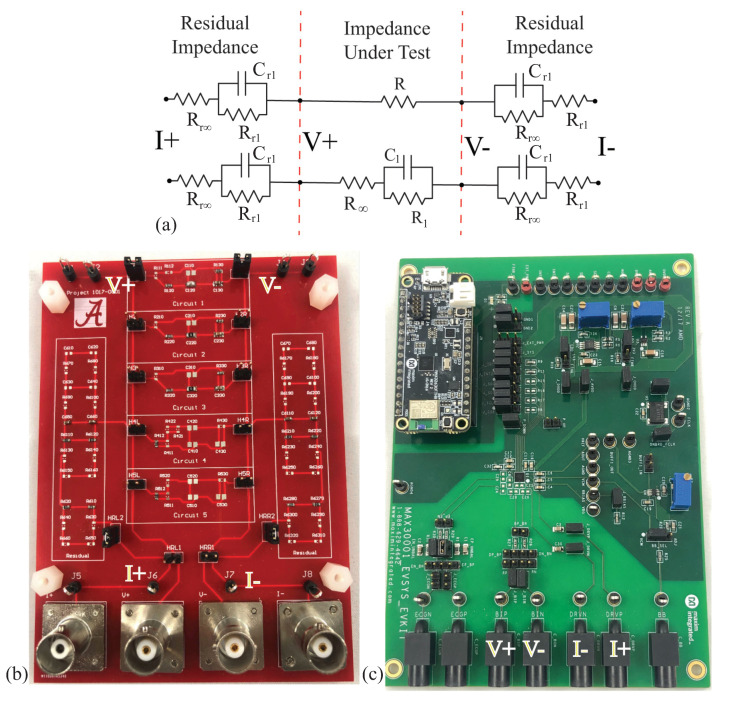
(**a**) Electrical schematic of topologies with series residuals, (**b**) realized using a PCB populated with discrete surface-mounted components, which was (**c**) measured using the MAX3000x to characterize the performance of this IC for measuring both resistance and reactance in the range representative of localized bioimpedance measurements.

All bioimpedance measurements require direct interfacing of the measurement device with the tissue under study. This is most often achieved using “wet” silver/silver-chloride (Ag/AgCl) gel electrodes [8,43], dry textile electrodes [21,44], or metal electrodes [45]. The use of electrodes introduces a series residual impedance between the device and tissue, referred to as the tissue/electrode interface impedance. The term residual is used in this work to describe the series impedance that is introduced by the test fixture, not the parasitic elements between the device and tissue under testing or external environment. This language is derived from the terminology in the Keysight documentation related to impedance measurements [46]. While using a tetrapolar electrode configuration reduces the impact of this impedance, it cannot eliminate it. For this reason, the discrete circuits that are realized in this work to emulate typical tissue impedance also include the electrode/tissue impedance between the current excitation pins and the circuit under measurement. A 2R−1C model, which is shown in Figure 4a, is utilized as the residual impedance to emulate the frequency-dependent impedance of the tissue/electrode interface. This model was previously utilized in [47,48]. The impedance of the 2R−1C model is given by:

(3) $$Z_{\mathrm{Res}}=R_{r\infty }+\frac{R_{r1}}{1+\left(j\omega \right)R_{r1}C_{r1}}$$

Because the presence of this impedance has been shown to degrade the performance of instruments, such as the Keysight E4990A, in previous research efforts [49], this work includes the electrode/tissue impedance to characterize the performance of the MAX3000x in situations that are representative of bioimpedance measurements.

To characterize the MAX3000x’s performance, impedance data were collected from two resistors and three 2R−1C models. These 2R−1C models represent the frequency-dependent behavior of biological tissues. They also share the same topology as the electrode/tissue impedance with the impedance given by (4). While both share the same topology, the specific component values for the electrode/tissue interface and localized tissues have very different ranges. To prevent confusion between the components selected for the residual impedance and biological tissue impedance, the biological tissue impedance will use the following notations:

(4) $$Z_{\mathrm{Tis}}=R_{\infty }+\frac{R_{1}}{1+\left(j\omega \right)R_{1}C_{1}}$$

The specific component values in this technical note were selected to represent localized tissues based on the reported ranges of localized bicep tissues [4,5] and electrode/tissue interface values based on the reported values from [47].

The two resistors utilized for the initial characterizations were R=51 Ω and R=110 Ω, and $Z_{\mathrm{Tis}}$ were: [$R_{\infty }$, $R_{1}$, $C_{1}$] = [62 Ω, 20 Ω, 0.47 µF], [20 Ω, 36 Ω, 0.047 µF], and [75 Ω, 36 Ω, 0.22 µF], which are referred to as Models 1, 2, and 3, respectively. The series residual impedances of the tissue/electrode interface were emulated using: $R_{r\infty }=200$ Ω, $R_{r1}=62$ kΩ, and $C_{r1}=0.011$ µF (note that the value of $R_{r1}$ is orders of magnitude higher than $R_{1}$). The two configurations of discrete components representing (i) a simple resistance and (ii) a localized tissue with series residuals are detailed in Figure 4a. A custom printed circuit board (PCB) populated with discrete surface-mounted components, jumpers for selecting the desired model (with five available per PCB), and connectors for interfacing with the test equipment was utilized to realize these circuits for testing. For reference, this PCB is provided in Figure 4b.

While the MAX3000x has a user-configurable excitation frequency (from approximately 125 Hz to 125 kHz, with slight variations based on internal clock settings), it is limited to measuring only a single frequency. There is no internal sweep function, but the device can be reconfigured during operation to collect different measurements. This was the approach utilized in this work to collect multi-frequency measurements. Each single-frequency impedance was measured for approximately 5 s after configuration of the development kit using Maxim’s provided tools. A more detailed analysis of the timing requirements for this configuration is outlined in Section 6.3. This set of values was saved as an ascii text file for later reduction to a single mean value during post-processing. The initial 0.3 s of data were omitted from this mean to prevent the settling after device configuration from impacting this value. For the measurements reported in the following sections, unless otherwise noted, the MAX3000x was configured to enable the bioimpedance channel measurements with: FMSTR = 01 (main clock settings), low-power mode, 80 *v*/*v* instrumentation amplifier gain, 62.5 Hz sampling, 8 µA excitation current, and digital filtering disabled. While the excitation currents from 8 to 96 µA can be configured, the 8 µA option is the only value available for the eight measured discrete frequencies ranging from 1 to 128 kHz (and this is why it was selected here). Higher excitation currents have a limited subset of frequencies that they can be configured for (e.g., 96 µA currents are only available for 18 to 128 kHz measurements).

The reference measurements of each test circuit were collected with a Keysight E4990A precision impedance analyzer. These reference measurements were collected without the presence of the emulated electrode/tissue impedance. The Keysight E4990A has a reported accuracy of 0.1% for measurements from 1 kHz to 1 MHz in the range from 10 Ω to 50 kΩ. This instrument was interfaced with the PCBs for measurement using the Keysight 16089D fixture and configured to collect measurements from eight discrete frequencies ranging from 1 to 128 kHz. For these measurements, a 1 mA current excitation was set, with no open/short/load compensation. While this excitation current is higher than the current utilized by the MAX3000x, this did not significantly impact the reference impedance values collected from the Keysight E4990A. To confirm, excitation currents of approximately 6 µA to 1 mA were utilized to collect measurements with <0.07 Ω difference between cases. The complete set of details for this validation is provided in Appendix A. The specific measured frequencies were selected to match those of the MAX3000x. Twenty measurements at each of the eight frequencies were collected and averaged to generate the reference values. While measurements at 125 Hz can be collected by the MAX3000x, this technical note is focused on the frequency range from 1 to 128 kHz because measurements within this range have been the focus of recent localized bioimpedance research (and this is the application area that this technical note aims to support). The frequencies and applications of recent localized bioimpedance research include the following: Nescolarde et al. utilized 50 kHz measurements in their studies focused on muscle injuries [2,3], Mabrouk et al. utilized 5 to 100 kHz measurements in their ankle edema assessment efforts [9], and Zhu et al. utilized 5 kHz to 1 MHz during their hydration efforts [7].

### 3.1. Resistance and Reactance Accuracy

As noted previously, it is hypothesized the MAX3000x utilizes quadrature demodulation with a single multiplication for the measurement of attached materials/devices. With a single multiplication, this IC should be capable of measuring both the resistance and reactance (though not simultaneously). To confirm this hypothesis and to assess the accuracy, resistance and reactance measurements from two resistor models and three 2R−1C models were collected and compared to the Keysight E4990A impedance measurements. The MAX30001 was configured to use phase offset adjustments of $0^{\circ }$ and $90^{\circ }$ for the resistance and reactance measurements, respectively.

**Figure 5:**
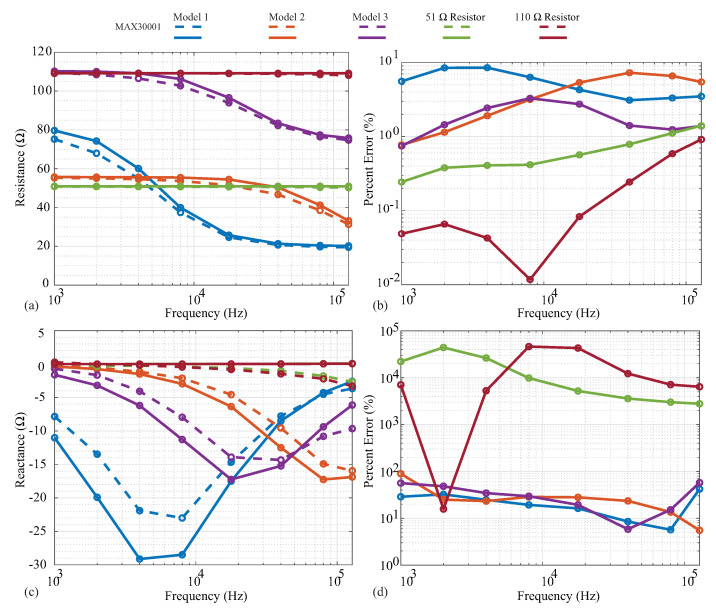
MAX3000x (dashed) and Keysight E4990A (solid) measurements of (**a**) resistance and (**c**) reactance of two resistors (51 Ω, 110 Ω) and three 2R−1C models. For comparison, the relative errors of the MAX3000x (**b**) resistance and (**d**) reactance compared to the Keysight E4990A data are provided.

The measured resistance and reactance are shown in Figure 5a,c, with the line colors indicating the specific cases and line styles indicating the measurement instruments. Dashed and solid lines represent the MAX3000x and Keysight E4990A measurements, respectively, with the circles representing the exact frequencies measured. Next, the relative errors of the MAX3000x measurements compared to the reference Keysight E4990A values are given in Figure 5b,d.

Both the 51 Ω and 110 Ω resistors display resistances that are constant with the frequency in Figure 5a, as expected. The MAX3000x measurements show very good visual agreement with the reference measurements, with <2% relative error across all frequencies in Figure 5b. Observing the MAX3000x resistances measured from the 2R−1C models, visual deviations from the reference measurements occur in the transition band from low to high frequency. This is the frequency band at which the resistance decreases from the ideal low-frequency value of $R_{\infty }+R_{1}$ towards ideal high-frequency value of $R_{1}$. The transition band is when the reactance of the 2R−1C model will have its largest value; this model approaches a reactance of zero at very low and very high frequencies. The deviations in resistance reach nearly 10% (compared to the reference measurements) for the individual frequencies measured of Models 1 and 3 in Figure 5b. With only the resistance measurements not exhibiting significant deviations (even with the residual impedance), this implies that the reactance component of the impedance being measured (and not the residual) has the greater effect on the resistance accuracy, thus degrading its performance. This should be considered when evaluating the MAX3000x for applications that require high accuracy.

The MAX3000x measurements collected using phase offset adjustments of $90^{\circ }$ for all of the discrete test circuits (with the residual series impedance) are given in Figure 5c as dashed lines. The reference measurements collected using the Keysight E4990A are given as solid lines. As expected, the 51 Ω and 110 Ω resistor reference measurements have 0 Ω reactances, though the MAX3000x measurements have visual deviations of reactances above 10 kHz. Observing the 2R−1C values, the reactance shape is correct for the MAX3000x measurements, but with a decreased magnitude in comparison to the reference values. The deviations for the 2R−1C models range from approximately 10% to 100% (with even higher errors for the resistor measurements, which are expected to be an artifact of the very low values to be measured for them). While these results do confirm that the MAX3000x does measure reactance when using phase offset adjustments of $90^{\circ }$, it has much lower accuracy compared to the resistance measurements collected in this work.

### 3.2. Relative Resistance Alterations

**Figure 6:**
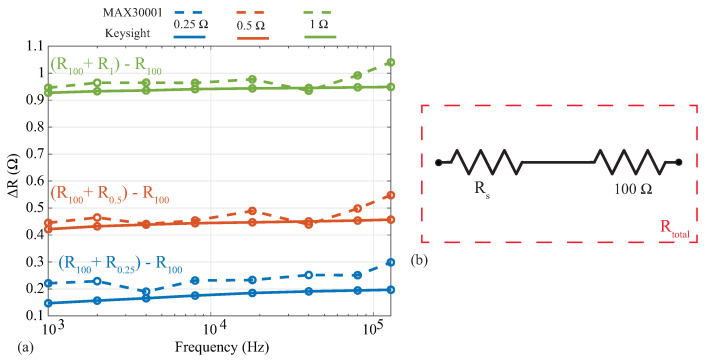
Absolute differences in resistance between (**a**) MAX3000x (dashed) and Keysight E4990A (solid) after addition of (**b**) 0.25 Ω, 0.5 Ω, and 1.0 Ω series resistors ($R_{s}$) to a 100 Ω resistor.

Analyses of localized bioimpedance have explored the relative alterations of the impedance with respect to determining an absolute value as an indicator of tissue health. As an example, Mabrouk [18] and Hersek [8] analyzed differences between impedance values, not the impedance magnitude, to assess ankle edema and knee joint health. In these applications, deviations in reported tissue values from an absolute reference may not impact the analysis of relative changes. To evaluate the MAX3000x’s resolution (and its suitability for applications utilizing relative changes), resistance measurements were collected from a 100 Ω resistor with additional series resistances of 0.25 Ω, 0.5 Ω, and 1.0 Ω. This configuration can be seen in Figure 6b, where the 0.25 Ω, 0.5 Ω, and 1.0 Ω resistors are $R_{s}$. Measurements of these three series resistance cases were collected using both the MAX3000x and Keysight E4990A. To assess the relative change in resistance as a result of the additional series resistance, the difference between each series-added case and the base 100 Ω was calculated ($\Delta R=R_{total}-R_{100\;\Omega }$). These results are shown in Figure 6, where the blue, red, and green lines correspond to the addition of 0.25 Ω, 0.5 Ω, and 1.0 Ω resistors, while the dashed and solid lines correspond to the MAX3000x and Keysight measurements, respectively. Note that the reference values from the Keysight E4990A support that there are mΩ level variations of these resistors across this frequency band. Comparing the MAX3000x measurements with the Keysight reference values, all are within 0.2 Ω. This level of resistance precision is similar to those of other sensing systems that have utilized the AD5933 and custom circuitry [9].

### 3.3. Analog High-Pass Filter Impact

**Figure 7:**
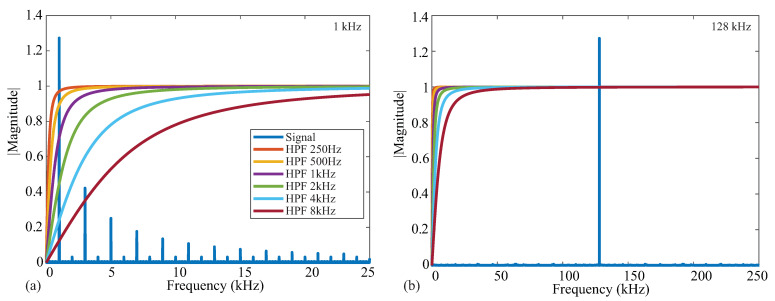
Frequency spectrum of (**a**) 1 kHz and (**b**) 128 kHz square wave signals in comparison to the magnitude response of high-pass filters with cutoff frequencies from 250 Hz to 8 kHz.

From the details in Figure 1a, recall that the on-board signal conditioning of the MAX3000x contains an analog high-pass filter prior to the input stage. This filter stage has programmable cutoff frequencies of 250 Hz, 500 Hz, 1 kHz, 2 kHz, 4 kHz, and 8 kHz. While this filter will remove low-frequency signal contributions from the voltage measured by the MAX3000x, it also has the potential to impact the impedance values calculated using this voltage. To characterize this impact on resistance and reactance, measurements were collected from the 2R−1C Model 1 with the high-pass filter configured to: bypass, 250 Hz, and 8 kHz. These represent the lowest and highest cutoff frequencies of this IC to compare with those of the bypass case. Both resistance and reactance values are given in Table 2. From these values, the measurements with the analog filter enabled are lower than the cases when the filter is not enabled, with greater impact on the 1 kHz value than the 128 kHz value—a result of the filter reducing the spectral content of the frequency of interest. Figure 7a,b illustrate this effect, plotting the magnitude response of ideal high-pass filters given by (1) for increasing cutoff frequencies (from 250 Hz to 8 kHz) against the spectral content of an ideal square wave with a magnitude of 1 at 1 and 128 kHz. Notice that the 1 kHz signal is within the stopband of most of the ideal responses in Figure 7a, supporting why it is significantly impacted as the filter cutoff frequency is altered. The 128 kHz signal in Figure 7b is in the pass-band regardless of the cutoff, which is why it is significantly less impacted by the filter. An interesting result of this exploration is that the reactance measurements in Table 2 have positive values with filtering enabled. As the test circuit has capacitive features in both the reference and MAX3000x measurements (without filtering), this implies that the reactance measurements are more significantly impacted by the filtering. Based on these results, it is recommended that the analog high-pass filter remains disabled for frequencies <80 kHz in applications that require the best accuracy with this IC.

### 3.4. Power Modes (Low Power, Low Noise)

**Figure 8:**
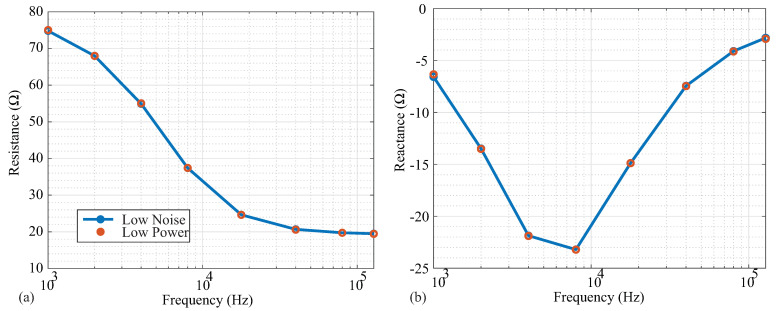
(**a**) Resistance and (**b**) reactance measurements of Model 1 by MAX3000x in low-noise (blue) and low-power (red) operating modes.

Following the high-pass filtering in Figure 1a, the voltage sensed by the MAX3000x is input into an instrumentation amplifier that has two available power modes: low power and low noise. While low-power modes are desired for wearable systems and are an attractive feature for the MAX3000x’s integration into wearable systems, it is also important to determine the potential trade-offs between these operating modes. To determine if there are differences in accuracy between these two modes, resistance and reactance data were collected from the 2R−1C Model 1. The measurements in both modes are given in Figure 8a,b. From a visual inspection, there was no deviation between the measurements in the different power modes for any of the measured frequencies. To further quantify the difference between power mode measurements, the standard deviations of the averaged impedance values at each frequency are given in Table 3. The low-noise measurements had lower standard deviations compared to the low-power measurements for all frequencies. However, this difference was relatively small, and may only impact applications requiring detection of impedance changes near the limit of the MAX3000x’s resolution.

### 3.5. Programmable Gain Amplifier

**Figure 9:**
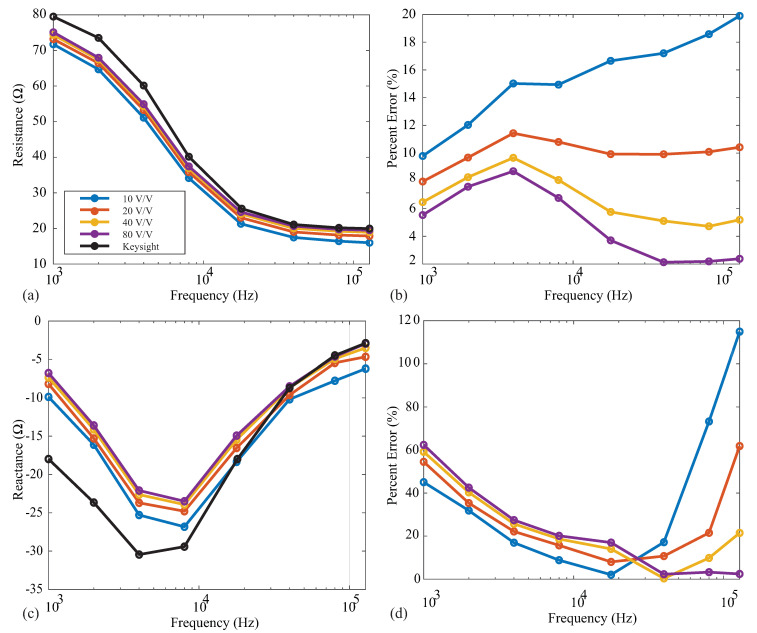
MAX3000x’s (**a**) resistance and reactance (**b**) measurements of 2R−1C Model 1 and relative (**c**) resistance and (**d**) reactance errors (compared to the Keysight E4990A reference values) using PGA configurations of 10 *v*/*v* (blue), 20 *v*/*v* (red), 40 *v*/*v* (orange), and 80 *v*/*v* (purple).

Following the instrumentation amplifier in the MAX3000x’s signal conditioning chain, there is a PGA prior to the digital conversion and filtering stages. This PGA has user-selectable gains of 10 *v*/*v*, 20 *v*/*v*, 40 *v*/*v*, and 80 *v*/*v*. The specific gain should be selected to take advantage of the complete ADC input range’s maximum resolution. To observe the effect of the gain on measurements that are representative of localized tissues, resistance and reactance measurements were collected from the 2R−1C model 1 using each of the gain settings. These measurements and their percent errors (compared to the reference values) are given in Figure 9a–d for the resistance and reactance, respectively. In each of these figures, the solid black line shows the reference Keysight E4990A values. For the resistance measurements, the percent error decreases as the gain increases for all frequencies. The most accurate resistance values were obtained using a gain of 80 *v*/*v*. Observing the 128 kHz resistances, the error decreased from 20% to <4% when the gain was increased from 10 *v*/*v* to 80 *v*/*v*. This is attributed to the higher gain supporting amplified voltages that take advantage of the ADC resolution. Notice from Figure 9 that the reactance values did not follow this same trend across all frequencies. The 10 *v*/*v* gain yielded the smallest error in these measurements for frequencies ≤40 kHz, while 80 *v*/*v* had the smallest percent error for high frequency values. These results continue to support that the MAX3000x may not be optimized for accurate reactance measurements.

### 3.6. Sampling Rate

After the final amplification by the PGA in the MAX3000x bioimpedance channel, the signal is converted by a ΣΔ ADC into a digital representation that can be sampled externally at approximately 32 or 64 sps. The sampling rate is dependent on the internal FMSTR configuration. Internally, the difference between sampling rates is the decimation ratio applied to the ΣΔ ADC, with a larger decimation ratio for lower sampling rates, which also increases the measurement latency. To determine if the sampling rate has an effect on the accuracy of the resistance and reactance data, these measurements were collected from the 2R−1C Model 1 using both settings. The standard deviations of the averaged values at each frequency are given in Table 4. In this case, the standard deviation is larger for the 64 sps configuration at all frequencies. However, similarly to the power-mode exploration, the difference as a result of the sampling was relatively small, and may only impact applications requiring the detection of impedance changes near the limit of the MAX3000x’s resolution.

## 4. Current Excitation Validation

**Figure 10:**
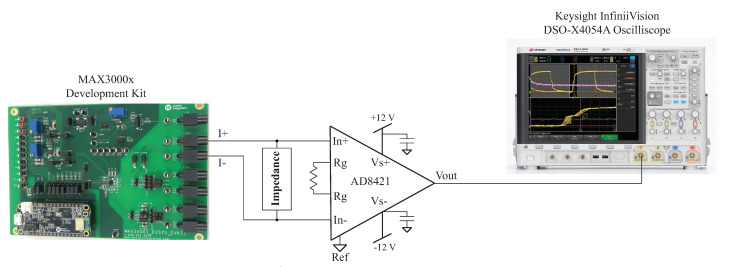
Experimental setup for measuring the excitation current applied by the MAX3000x development kit to a test impedance.

**Figure 11:**
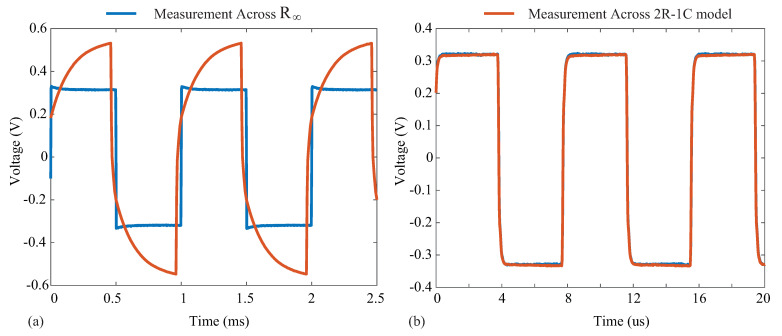
Voltage waveform collected using a Keysight DSO-X4054A oscilloscope in the setup of Figure 10 to measure voltage across $R_{\infty }$ (blue line) and the complete 2R−1C model (orange line) at (**a**) 1 kHz and (**b**) 128 kHz.

One source of error from the impedance calculations using (2) in the post-processing of the MAX3000x’s reported measurements is the assumption that the applied excitation current and programmed current are equal. That is, in reference to the previous calculations in this work, the excitation current is exactly 8 µA. Any deviations from this ideal value degrade the accuracy of the calculated impedance. While the MAX3000x’s documentation details a ±30% accuracy across all temperature ranges using an internal bias resistor and a ±10% accuracy using an external bias resistor, the specific current for the test setup in this work was assessed for comparison against these values. The excitation current generated by the MAX3000x was measured using the test configuration given in Figure 10. In this setup, an instrumentation amplifier (AD8421 from Analog Devices) was connected across the same load impedance connected to the MAX3000x development kit. The gain of the instrumentation amplifier in this setup is given by:

(5) $$\mathrm{Gain}=1+\frac{9.9\;k\Omega }{R}$$

where *R* is an external resistor connected across the $R_{G}$ pins on the AD8421. For the testing configuration in this work, R=99.2 Ω, yielding a gain of 100.78 using (5). Supply voltages of ±12 V were generated by a Keysight E3631A power supply for the instrumentation amplifier. Measurements from the instrumentation amplifier were collected using a Keysight DSO-X4054A oscilloscope when excitation currents of 8 µA were applied to the resistive loads and a 2R−1C model. Samples of the voltage waveforms for a 1 and 128 kHz excitation applied to the 2R−1C model are given in Figure 11. In Figure 11a,b, the blue waveform represents the voltage across $R_{\infty }$ and the orange waveform represents the voltage across the 2R−1C model. Notice that at 1 kHz, the voltage across the 2R−1C model is dominated by the capacitive characteristics at this frequency (seen as the charging/discharging voltage), and at 128 kHz, the response is dominated by the resistive characteristics (and this is why the voltages across the model and $R_{\infty }$ visually align). The excitation current ($I_{\mathrm{Ex}}$) was calculated from the measured waveform with:

(6) $$I_{\mathrm{Ex}}=\frac{V_{\mathrm{Ex}}}{R_{\mathrm{DUT}}}$$

where $V_{\mathrm{Ex}}$ is the amplitude of the voltage induced by the current and $R_{\mathrm{DUT}}$ is the resistance of the device under testing. Note that in the 2R−1C measurements, the voltage used for this calculation was the value across $R_{\infty }$.

Additionally, the MAX30001 has four different current modes, with a datasheet recommendation of using the “chopped w/o LPF”, where LPF refers to a low-pass filter, which is a mode for impedance applications. To assess if the current excitation mode impacts the excitation current, measurements were collected in each. The MAX3000x excitation currents (in all modes) calculated from measurements of a 502.8 Ω resistor and a 2R−1C model using (6) are given in Table 5. In the case of the 2R−1C model, the voltage was measured across $R_{\infty }$, which had measured Keysight E4990A values of 385.48 Ω and 385.59 Ω at 1 and 128 kHz, respectively. For measurements of both models, the excitation current had deviations <5%, with higher deviations for the 128 kHz calculations. This confirms that the excitation current of the MAX3000x is within the expected operating range, but also that the deviation from the ideal value will increase the error of the calculated impedance values (proportional to this error amount). There was not a significant deviation of the current based on the mode for this series of measurements. Previous efforts have explored a two-point calibration to reduce resistance errors, decreasing resistance errors to <0.22% (though similar improvements were not reported for reactance) [30]. The use of multiple impedance loads coupled with a multivariate linear regression algorithm proposed by Mabrouk et al. [18] may also improve the accuracy of MAX3000x measurements and warrants further investigation and implementation.

## 5. MAX3000x Application Support

The previous sections detailed how the configuration settings of the MAX3000x impact the accuracy of the resistance and reactance measurements collected by this IC. Beyond the accuracy, factors such as measurement range and available signal conditioning are important when evaluating this device for a specific application. In support of understanding additional features of this IC, limits on the range of resistances that can be measured and internal electrode detection functionality are explored.

### 5.1. Saturation Calculation

Previously, the 80 *v*/*v* PGA gain yielded the resistance measurements with the highest accuracy (in the subset of conditions investigated) and was recommended for taking advantage of the ADC resolution. This recommendation assumes the MAX3000x’s sensed voltage does not cause saturation of the signal conditioning channel. The differential input of the sensing pins is not recommended to exceed 90 mV${}_{\mathrm{pp}}$, which provides guidance for the maximum impedance range that can be measured for each configuration. To prevent saturation, the differential voltage to the ADC must be less than the reference voltage (1 V), which requires that:

(7) $$V_{\mathrm{ADC}}=Z_{M}\times I_{\mathrm{Ex}}\times G\leq 1\;V$$

where $Z_{M}$ is the impedance to be measured by the MAX3000x, $I_{\mathrm{Ex}}$ is excitation current (8 µA, 16 µA, 32 µA, 48 µA, 64 µA, 80 µA, 96 µA), and *G* is the PGA gain (10 *v*/*v*, 20 *v*/*v*, 40 *v*/*v*, 80 *v*/*v*). The saturation condition given by (7) can be re-arranged to yield the theoretical limit of the impedance that can be measured for a particular configuration:

(8) $$Z_{M}\leq \frac{1\;V}{I_{\mathrm{Ex}}\times G}$$

As an example, the configuration settings utilized throughout this work (8 µA, 80 *v*/*v*) yield a maximum impedance of $Z_{M}<1562.5$ Ω using (8), which is above the range of reported localized bioimpedance measurements.

### 5.2. Electrode Detection

**Figure 12:**
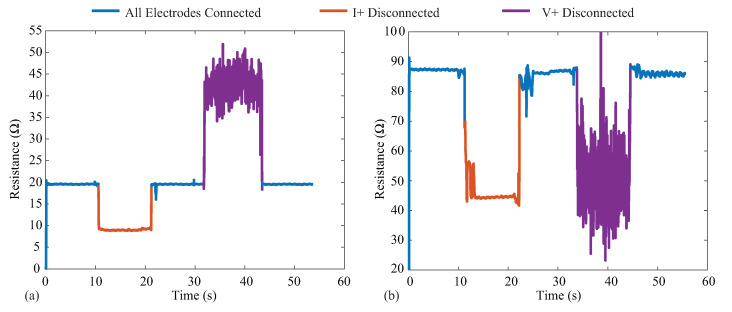
Resistance measurements at 128 kHz with current and voltage electrode disconnection events on the (**a**) testboard and (**b**) the bicep. The blue corresponds to times when all electrodes are connected, orange represents when the *I+* current lead is disconnected, and purple represents when the *V+* voltage lead is disconnected.

The MAX3000x provides three lead-off/electrode detection methods using digital and analog threshold comparisons, which alter internal register flags on the IC for monitoring by the controlling device. Electrode detection is critical for wearable bioimpedance applications, where movement can cause electrode disconnection events that may go unnoticed by the user and degrade both the quantity and quality of the collected measurements. Identification of the connection status of the electrode could be used in both real-time biofeedback (e.g., indications to the user to check the electrodes), real-time system optimization (maintaining low-power operation without data collection until electrodes are connected), or in data post-processing (e.g., removal of data collected during disconnection events from analysis). To visualize the impact that electrode disconnects have on the resistance data reported by the MAX3000x, 128 kHz data were collected from two test cases (PCB test board and bicep tissue) for 60 s. For each case, a tetrapolar configuration was utilized, with the cable connected to an electrode disconnected at the 10 and 30 second timepoints and re-attached at the 20 and 40 second timepoints, respectively. Figure 12a,b illustrates the data collected from the 2R−1C model on the test PCB and from the bicep tissue of a participant. In each figure, the blue line corresponds to times when all electrodes are connected, orange represents when the *I+* current lead is disconnected, and purple represents when the *V+* voltage lead is disconnected. It is important to observe that both disconnection events yielded resistances that were within the range previously reported for localized bioimpedance measurements [4,5]. This highlights that these types of events could significantly impact the interpretation of localized bioimpedance data. While a significant change in continuous resistance data can serve as a marker of an electrode disconnection event, if multi-frequency measurements are collected, this event may not be captured (this limitation is discussed further in Section 6.3). Additionally, the voltage disconnection event has high variability, which has also been observed with activity and muscle contraction (discussed further in Section 6.2). This highlights the importance of electrode detection capabilities, whether implemented in hardware or in post-processing algorithms for wearable systems that collect localized bioimpedance data for accurate interpretation.

One on-chip electrode disconnect approach of the MAX3000x implements analog current monitoring, which detects when the voltages on the driving pins are outside of a programmed operating range. Another on-chip method uses a digital lead-off detection to monitor the output of the ADC and compares the value to a threshold defined by the user. To determine the threshold needed for a specific application, the following equation can be used:

(9) $$\mathrm{Threshold}=Z_{\mathrm{measured}}\times 2^{19}\times \mathrm{CG}\_\mathrm{MAG}\times \mathrm{GAIN}$$

where $Z_{measured}$ is the expected impedance value to be measured by the MAX3000x, CG_MAG is the current magnitude, and GAIN is the gain setting. While this methodology can be used if battery life is of concern, it is also important to note this can also be replicated in post-processing. The final lead-off detection on-chip capability of the MAX3000x sinks/sources a DC current on the BIP/BIN pins and utilizes a comparator with a specified threshold to detect a lead-off condition. The setting, however, requires a disconnection event of ≥115 ms to trigger a disconnect flag (which may limit this detection method to single-frequency, continuous measurements). Together, these methods can be utilized to provide additional information to evaluate if electrode disconnection events have occurred and to take the necessary action (in terms of reporting or post-processing), increasing the usability of the MAX3000x for wearable applications with limited user focus on the electrode connection quality.

## 6. Example Application: Bicep Bioimpedance During Exercise

One recent application of localized bioimpedance measurements explored the monitoring of tissues during activity to identify indicators of fatigue and, potentially, injury [4,5]. To demonstrate the functionality of the MAX3000x specific to this application, measurements of localized bicep tissues were collected throughout an activity protocol in which a participant (female, 25 years old) completed repetitions of dumbbell bicep curls using two different weights (10 and 15 lbs).

**Figure 13:**
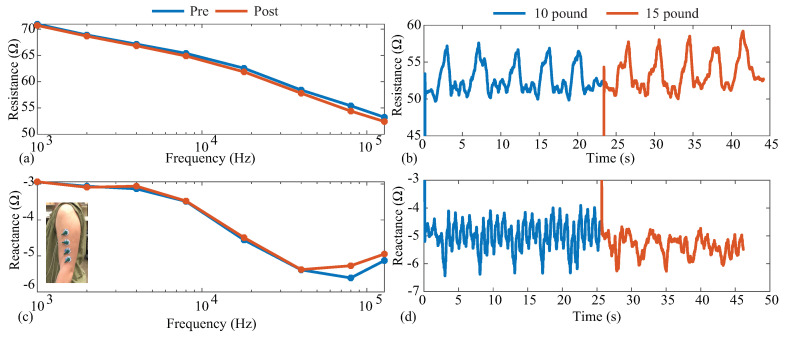
(**a**,**b**) Resistance and (**c**,**d**) reactance measurements of localized bicep tissues collected before/after (multi-frequency, 1 to 128 kHz) and throughout (single frequency, 128 kHz) an activity protocol of dumbbell bicep repetitions.

For this data collection, a tetrapolar configuration of Ag/AgCl electrodes was placed on the bicep of a single participant after cleaning the site with alcohol wipes. The electrode placement on the biceps is given in the subset of Figure 13c. During this test, multi-frequency (1 to 128 kHz) measurements were collected immediately before and after activity, and single-frequency (128 kHz) measurements were collected throughout the activity. Because the MAX3000x cannot collect resistance and reactance simultaneously, resistance data were collected prior to reactance at all stages, with reconfiguration of the MAX3000x required for each change in frequency or measurement type (resistance/reactance). Other than changes in the frequency and measurement type, the MAX3000x was configured with the following settings: filtering disabled, 8 µA current amplitude, 80 *v*/*v* gain, 64 sps.

### 6.1. Activity Protocol

The summarized activity protocol steps and measurements are detailed below:

(1) Multi-frequency (1 to 128 kHz) resistance and then reactance were collected prior to activity while the participant was standing with arms relaxed, resting naturally at the sides of the body.
(2) The 128 kHz resistance was collected continuously during five repetitions of 10 lbs over approximately 25 s;
(3) The 128 kHz reactance was collected continuously during five repetitions of 10 lbs over approximately 25 s;
(4) The 128 kHz resistance was collected continuously during five repetitions of 15 lbs over approximately 25 s;
(5) The 128 kHz reactance was collected continuously during five repetitions of 15 lbs over approximately 25 s;
(6) Multi-frequency (1 to 128 kHz) measurements of resistance and then reactance were collected post-activity while the participant was standing with arms relaxed, resting naturally at the sides of body.

### 6.2. Activity Protocol Results

The pre- and post-exercise multi-frequency measurements are given in Figure 13a,c for the resistance and reactance, respectively. The blue and red lines represent the pre-activity and post-activity measurements, respectively. It can be observed that the resistance decreased with increasing frequency, decreasing from approximately 70 Ω at 1 kHz to 53 Ω at 128 kHz. The experimental tissue reactance in Figure 13c follows the same reactance trends as the 2R−1C model in Figure 5c. The trends and ranges of both resistance and reactance aligned with previous reports of tissue resistance [4,5] and supported the earlier use of the 2R−1C model in this work to emulate the tissue impedance. Comparing the pre- and post-activity resistance, there was a slight decrease across all frequencies. Specifically, 128 kHz had a 0.8 Ω decrease in resistance and a 0.18 Ω increase in reactance for the post-activity measurements compared to the pre-activity values. Decreases in bicep resistance were reported by Fu and Freeborn [4,50], which were attributed to increases in localized fluid at the tissue site. The decreases in this work were lower than those reported by Fu and Freeborn, which is attributed to the lower exercise stimulus utilized here. Only a total of 20 repetitions were completed in this protocol.

The 128 kHz resistance and reactance collected throughout the protocol are combined in Figure 13b,d so that data from repetitions at different weights are included in the same figure. In both figures, the blue data represent measurements collected when a 10 lb dumbbell was curled, and red represents when a 15 lb dumbbell was curled. The initial increases at 0 s and approximately 25 s were the periods in which the MAX3000x data settled after being configured. Observing both the resistance and reactance, it is clear that the activity impacted the bicep tissues and the resulting bioimpedance. Observing the resistance in Figure 13, the 128 kHz data increased during the periods of (expected) muscle contraction, reaching average peaks of 56.97 Ω and 58.10 Ω compared to the approximated 50 Ω baseline. These results align with those reported by Kitchin and Freeborn, detailing acceleration and impedance profiles during bicep curls [51], with a similar resistance profile in the Keysight E4990A datasets. The results of both multi-frequency and single-frequency data here highlight that the MAX3000x IC can measure both the resistance and reactance of localized tissues during activity, which could support further research into tissue changes during free-living activity if integrated into a wearable device.

### 6.3. Frequency Sweep Limitations

**Figure 14:**
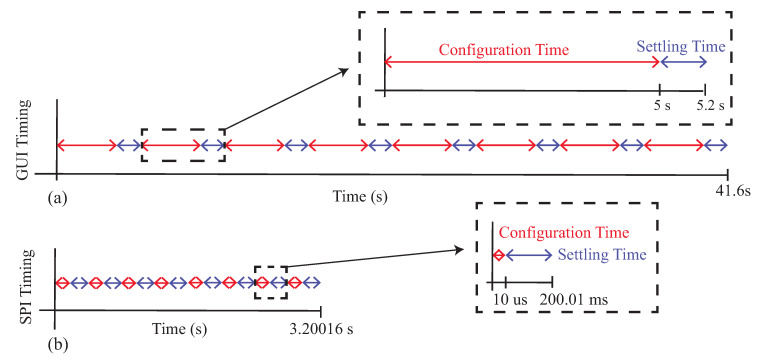
Timing diagram for MAX3000x multi-frequency impedance measurements using (**a**) a manual GUI configuration and (**b**) a microcontroller configuration.

While the MAX3000x is able to measure the resistance and reactance of localized tissues, the time needed to reconfigure the type of measurement and frequency impacts multi-frequency measurements and their interpretation. That is, they cannot be interpreted as being collected simultaneously and being an instantaneous representation of the tissue. To highlight this, Figure 14 illustrates the approximated minimum timing diagrams for two cases: (1) manually configuring the MAX30001EVSYS using the software on a computer and (2) configuring the MAX3000x using SPI communication from a microcontroller (or similar device). These timing diagrams are representative of the measurements collected in this study (manual configuration) and cases when the MAX3000x is integrated into a wearable system. In these timing diagrams, multi-frequency measurements at eight frequencies of either resistance or reactance were assumed, where each frequency was given a configuration and settling time. A settling time of 200 ms was used (which is larger than the approximately 100 ms reported in the datasheet as the fastest case for certain configurations). For the manual configurations, 5 s were used as the approximation for this process (based on the earlier experiences during data collection for this study). A single measurement at this point required less than 10 ms to collect, an order of magnitude below the other features, and this was neglected from the total timing. Combining these timings in Figure 14a, the approximate time to collect a multi-frequency sweep was 41.6 s, which increased to 83.2 s if both resistance and reactance data are collected.

It is important to clarify that while the multi-frequency measurements of Figure 13 are visualized as a single sweep, this period of time could capture events (e.g., contraction during activity) that may degrade the comparison of sweeps at different timepoints. Even if the time needed to configure the MAX3000x is reduced, it will still require a few seconds of time to collect a frequency sweep. For example, by using a microcontroller to configure the 10 MAX30001 registers for bioimpedance measurements, assuming a 3 MHz SPI clock, the configuration time could be reduced to 3.2 s for an eight-frequency sweep or 6.4 s if both the resistance and reactance are measured. This decrease is illustrated in Figure 14b. Compared to Figure 14a, the total time is dominated by the settling time between configurations (which cannot be reduced). Additionally, this estimation does not include the time required to convert and store raw measurements read from the MAX3000x FIFO, which could introduce additional time. While measurements coordinated by a microcontroller will decrease the total sweep time, multi-frequency measurements will still be susceptible to contraction or activity artifacts. This should be considered when evaluating data collected from this sensor.

## 7. Conclusions

The MAX3000x integrated circuit provides a small-form-factor, single-chip solution for the measurement of single- and multi-frequency bioimpedance (resistance and reactance) with significant on-chip signal conditioning options. Measurements of discrete impedances that are representative of localized tissue bioimpedance (20 Ω < *R* < 120 Ω, X<−30 Ω) and conditions (electrode/tissue interface impedance present) show that this IC has a relative error (without any calibration or compensation) of <2% for resistance-only measurements and <10% for the resistance component of complex impedance measurements. Measurements that included small changes in resistance (0.25 Ω) were also collected and confirmed the MAX3000x’s functionality in measuring relative mΩ resistance changes. Though the reactance measurement functionality of this IC was validated, the accuracy was lower than the resistance functionality. The on-chip power modes and sampling configurations did not have a significant impact on the reported measurements, but the filtering capabilities did degrade the accuracy of measurements below 80 kHz. Multi-frequency measurements are possible and were validated with this IC, but should be limited to applications in which changes are expected across minutes or hours but not milliseconds or seconds due to the reconfiguration and settling times that are necessary for implementing this functionality. While the MAX3000x does have performance limitations, it is still an attractive option for wearable applications focused on very small form factors.

## Figures and Tables

**Table 1:** Comparison of integrated circuits with bioimpedance functionality.

Feature	Integrated Circuit			
	AD5933	AFE4300	ADuCM350	MAX3000x
Freq. Range	1 kHz–100 kHz	8 kHz–64 kHz	80 Hz–75 kHz	125 Hz–131 kHz
Imp. Range	1 kΩ–10 MΩ	-	-	<12.5 kΩ
Lit. Imp. Range	R: 0–10 MΩ	R: 0–700 Ω	|Z|:50–3.4 kΩ	R: 0–200 Ω
	X: 0–100 Ω	X: 0–225 Ω	-	X: 0–20 Ω
Elect. Config.	Bipolar	Tetrapolar	Bipolar/Tetrapolar	Bipolar/Tetrapolar
Excitation	Sinusoidal	Sinusoidal	Sinusoidal/Trapezoidal	Square
Meas. Type	DFT	Demodualtion	DFT	Demodulation
Size	27 ${\mathrm{mm}}^{2}$	144 ${\mathrm{mm}}^{2}$	64 ${\mathrm{mm}}^{2}$	8 ${\mathrm{mm}}^{2}$
Release Year	2005	2012	2014	2019
AFE	Expand imp.	-	-	-
	range/tetrapolar			
Application	Variety	Body Comp. Analysis	Body Comp. Analysis	-
Literature	[18,20,21,22,23,24,25,26]	[32,33,34,35,36]	[37,38]	[30,31].

**Table 2:** Resistance and reactance measured in the 2R−1C model at 1 and 128 kHz with and without MAX3000x high-pass filtering functionality.

	HPF Setting					
Frequency	Bypass	250 Hz	8 kHz	Bypass	250 Hz	8 kHz
	Resistance			Reactance		
1 kHz	75.25 Ω	64.59 Ω	5.52 Ω	−6.41 Ω	−6.38 Ω	−6.76 Ω
128 kHz	19.48 Ω	18.78 Ω	18.55 Ω	−2.92 Ω	−2.50 Ω	−1.71 Ω

**Table 3:** Standard deviation of resistance and reactance measurements of the 2R−1C model by MAX3000x in low-power and low-noise operating modes.

	Frequency							
Power Mode	1 kHz	2 kHz	4 kHz	8 kHz	18 kHz	40 kHz	80 kHz	128 kHz
	Resistance							
Low Noise	0.028	0.024	0.032	0.024	0.023	0.036	0.043	0.024
Low Power	0.030	0.032	0.032	0.032	0.027	0.053	0.116	0.033
	Reactance							
Low Noise	0.031	0.032	0.030	0.032	0.032	0.084	0.070	0.051
Low Power	0.033	0.034	0.033	0.035	0.037	0.060	0.067	0.055

**Table 4:** Standard deviation of the resistance and reactance measurements of the 2R−1C model by the MAX3000x in the 32 and 64 sps sampling modes.

	Frequency							
Sampling Rate	1 kHz	2 kHz	4 kHz	8 kHz	18 kHz	40 kHz	80 kHz	128 kHz
	Resistance							
32 sps	0.020	0.023	0.025	0.020	0.023	0.023	0.100	0.030
64 sps	0.035	0.027	0.042	0.028	0.030	0.085	0.135	0.033
	Reactance							
32 sps	0.021	0.047	0.025	0.025	0.030	0.057	0.045	0.038
64 sps	0.033	0.036	0.033	0.035	0.037	0.059	0.067	0.055

**Table 5:** Measured stimulus current modes of the MAX30001. Mode 1: unchopped w/ LPF, Mode 2: chopped w/o LPF, Mode 3: chopped w/LPF, and Mode 4: chopped w/ resistive CM.

Resistor Model							
		**1 kHz**			**128 kHz**		
**Mode**	**Gain**	**Load (Ω)**	**Current (µA)**	**Percent Error (%)**	**Load (Ω)**	**Current (µA)**	**Percent Error (%)**
1	100.78	502.8	8.062	0.770	502.8	8.358	4.471
2	100.78	502.8	8.141	1.757	502.8	8.358	4.471
3	100.78	502.8	8.062	0.770	502.8	8.358	4.471
4	100.78	502.8	8.062	0.770	502.8	8.358	4.471
**2R−1C Model**							
		**1 kHz**			**128 kHz**		
**Mode**	**Gain**	**Load (Ω)**	**Current (µA)**	**Percent Error (%)**	**Load (Ω)**	**Current (µA)**	**Percent Error (%)**
1	100.78	385.48	8.19	2.28	385.59	8.38	4.70
2	100.78	385.48	8.17	2.16	385.59	8.27	3.42
3	100.78	385.48	8.17	2.10	385.59	8.38	4.70
4	100.78	385.48	8.17	2.10	385.59	8.38	4.70

**Figure A1:**
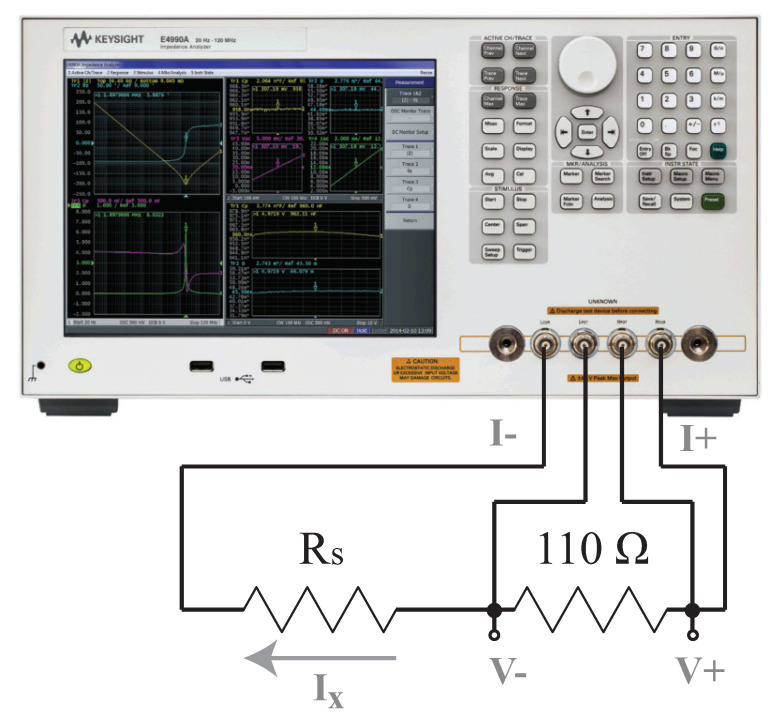
Test setup for limiting the Keysight E4990A excitation current with a series residual resistor ($R_{s}$) during the measurement of a 110 Ω resistor.

**Figure A2:**
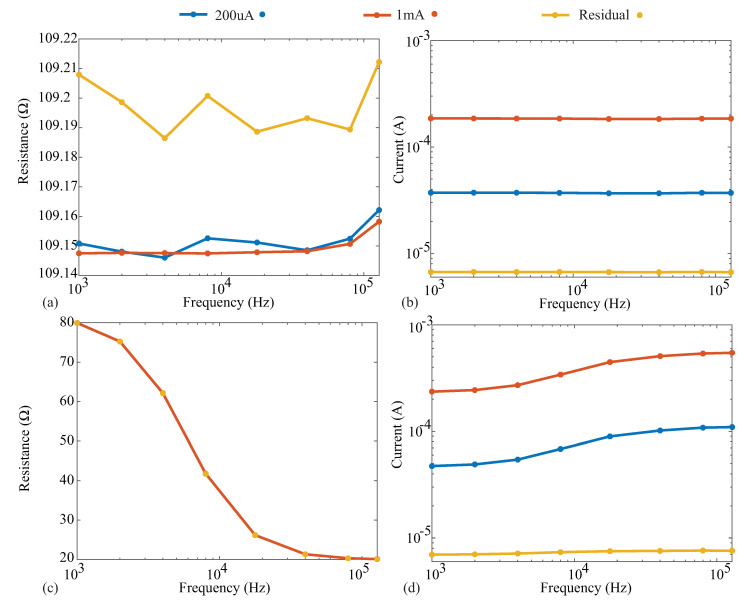
(**a**) Reported resistance and (**b**) injection current during measurement of a 110 Ω resistor when the Keysight E4990A was configured for 1 mA, 200 µA, and approximately 8 µA (using a current-limiting resistor); (**c**) reported resistance and (**d**) injection current during measurement of the 2R−1C Model 1 when the Keysight E4990A was configured for 1 mA, 200 µA, and approximately 8 µA (using a current-limiting resistor).

**Figure A3:**
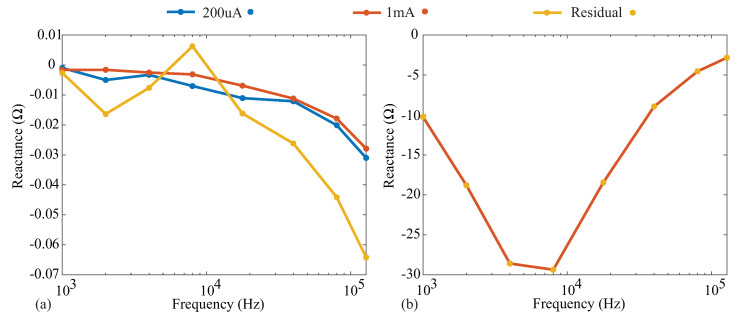
(**a**) Measured reactance of the 110 Ω resistor and (**b**) 2R−1C Model 1 when the Keysight E4990A was configured for 1 mA, 200 µA, and approximately 8 µA (using a current-limiting resistor).

## Data Availability

The data presented in this study are available on request from the corresponding author.

## Appendix A. Keysight E4990A Excitation Current Effects

To validate that the excitation current of the Keysight E4990A did not have a significant effect on the generated impedance measurements utilized as the reference values in this technical note, impedance measurements of a resistor and 2R−1C model were collected under different excitation values. Measurements of a 110 Ω resistor and 2R−1C model ($R_{\infty }=62$ Ω, $R_{1}=20$ Ω, $C_{1}=0.47$ µF, previously referred to as Model 1 in this work) were collected using the Keysight E4990A with excitation currents of 1 mA, 200 µA, and 6 µA. The 1 mA and 200 µA excitation currents were set by programming these values on the Keysight E4990A, while the 6 µA value was set through the addition of a current-limiting resistor in series with the impedance to be measured. A sample of this test configuration (with the current-limiting resistor) is given in Figure A1. The Keysight E4990A has a minimum excitation voltage (5 mV) that can be programmed for measurements. At this excitation, greater impedance values in series with the excitation source cause lower applied currents. The appropriate value of the current-limiting resistor (or series residual) for achieving an excitation current within the range of the 8 µA utilized by the MAX3000x was estimated using:

(A1) $$R_{s}=\frac{5\;\mathrm{mV}}{I_{x}}$$

where 5 mV is the minimum excitation voltage of the Keysight E4990A, $I_{x}$ is the desired injection current, and $R_{s}$ is the series residual resistance needed to achieve $I_{x}$. This yielded $R_{s}=625$ Ω, but based on the availability of discrete components, a single 610 Ω was used for further measurements.

The resistance and reactance measurements of the 110 Ω resistor and 2R−1C Model 1 are given in subplots a and c of Figure A2 and Figure A3, respectively. Additionally, the current excitations applied by the Keysight E4990A for all cases are also given in subplots b and d of these figures. Comparing the excitation currents for the 110 Ω measurements in Figure A2b, we notice that the 1 mA and 200 µA cases have reported currents of 184 and 37 µA (for the 128 kHz resistor case). Both are below their programmed values as a result of the load of the impedance to be measured. This does not impact the reported measurements because the Keysight E4990A uses the actual current excitation as opposed to the programmed value.

It can be observed for the 110 Ω resistor measurements, which are shown in Figure A2a, that even as the current decreases, there is minimal change in the measured resistance value. The absolute difference between the 1 mA measurements and those with 200 µA is <0.01 Ω. While the absolute difference between the 1 mA measurements and the residual case is slightly larger, it is still ≤0.06 Ω across the frequencies measured. Similarly, for the 2R−1C Model 1, which is shown in Figure A2c,d, the absolute differences between the 1 mA case and the 200 µA and residual injection current cases are <0.02 Ω and <0.07 Ω, respectively.

Similar trends were also observed through the comparison of the reactance measurements collected in each case. These measurements are given in Figure A3a,b for the 110 Ω resistor and 2R−1C Model 1. The absolute differences of the 200 µA case and residual injection current cases with respect to the 1 mA case are <0.005 Ω and <0.04 Ω for the resistor model and <0.015 Ω and <0.025 Ω for the 2R−1C Model 1, respectively. These results illustrate that varying the injection currents in the Keysight E4990A does not significantly impact the obtained resistance and reactance reference values in the frequency band of interest (1 to 128 kHz) utilized in this technical note. This supports the use of the generated 1 mA reference values for comparison against the MAX3000x values, even though the excitation current for the MAX3000x was much lower in magnitude.
